# Long-Term Blackcurrant Supplementation Modified Gut Microbiome Profiles in Mice in an Age-Dependent Manner: An Exploratory Study

**DOI:** 10.3390/nu12020290

**Published:** 2020-01-21

**Authors:** Lei Cao, Sang Gil Lee, Melissa M. Melough, Junichi R. Sakaki, Kendra R. Maas, Sung I. Koo, Ock K. Chun

**Affiliations:** 1 Institute of Marine Life Sciences, Pukyong National University, Busan 48513, Korea; 2 Department of Food Science and Nutrition, Pukyong National University, Busan 48513, Korea; 3 Department of Nutritional Sciences, University of Connecticut, Storrs, CT 06269, USA; Melissa.melough@uconn.edu (M.M.M.);; 4 Microbial Analysis, Resources and Services (MARS), University of Connecticut, Storrs, CT 06269, USA

**Keywords:** blackcurrant, gut, microbiome, mice, aging

## Abstract

Recent studies have suggested that blackcurrant (BC) anthocyanins have promising health benefits, possibly through regulating gut microbiome. Three- and eighteen-month old female mice were fed standard mouse diets for 4 months, each with or without BC (1% *w/w*) supplementation (*n* = 3 in each treatment group, 12 in total). We then assessed gut microbiome profiles using 16S sequencing of their feces. Old mice had a less diverse microbiome community compared to young mice and there was a remarkable age-related difference in microbiome composition in the beta diversity analysis. BC supplementation did not significantly affect alpha or beta diversity. The relative abundance of several phyla, including Firmicutes, Bacteroidetes, Proteobacteria and Tenericutes, was lower in old mice. BC downregulated Firmicutes abundance in young mice and upregulated Bacteroidetes in both age groups, leading to a decreased Firmicutes/Bacteroidetes ratio. There were age-specific differences in the effect of BC supplementation on the microbiome. Twenty-four operational taxonomic units showed a significant interaction between age and BC supplementation (*p* < 0.01), which suggests that the ecosystem and the host health status affect the functions and efficiency of BC intake. These results indicate that BC supplementation favorably modulates gut microbiome, but there are distinct age-specific differences. Studies with human hosts are needed to better understand BC’s regulatory effects on the gut microbiome.

## 1. Introduction

Blackcurrant (*Ribes nigrum*, BC) has antioxidant and anti-inflammatory properties that aid in the protection against cardiovascular and neurodegenerative diseases as well as diabetes [1,2]. Additionally, BC’s modulation of microbial populations has recently garnered attention: an in vitro study demonstrated that BC extract suppressed the growth of *E. coli* strains [3]; in rats, BC extract stimulated the growth of bifidobacteria and suppressed the growth of bacteroides and clostridia [4].

Anthocyanins are a group of major bioactive compounds of BC and may be the key regulator of its microbial-modulating properties. In particular, diglycosidic anthocyanins, including delphinidin-3-rutinoside and cyanidin-3-rutinoside, are the major anthocyanins constituents in BC [5]. Anthocyanins belong to the polyphenolic class of flavonoids and are glycosides of polyhydroxy and polymethoxy derivatives of 2-phenylbenzopyrylium or flavylium salt [6]. Anthocyanins are distinguished by the number of hydroxyl groups they possess, the degree of methylation of these hydroxyl groups, and the nature, number and position of sugar moieties attached, as well as the nature and number of aliphatic or aromatic acids attached to the sugars [6]. The majority of dietary anthocyanins are not absorbed at the upper gastrointestinal level so the unabsorbed anthocyanins reach the intestinal microbiome where they are metabolized and absorbed [7] via cleavage of glycosidic linkages and breakdown of anthocyanidin heterocycle [8]. This promotes the colonization of bacteria containing the requisite enzymes such as β-glucosidase that are necessary to catalyze the cleavage and breakdown reactions of anthocyanins [7]. In particular, mono- and diglycosidic anthocyanins are major targets of colonic microbiota [9].

Anthocyanins partly exert their health benefits through modulation of the gut microbiome. Specifically, the anti-inflammatory effect of anthocyanins may involve their interaction with the local microbiome and their functions in the intestine [10]. One study found that BC supplementation improved glucose metabolism only in diet-induced obese mice with an intact gut microbiome and not in mice with a disrupted gut microbiome [11], lending support to the notion that the gut microbiome plays a key role in mediating anthocyanins’ anti-inflammatory properties. Thus, the potential interactions between BC and the gut microbiome might be important to the overall health of their hosts.

It is now increasingly evident that young and aged hosts have different gut microbiome profiles [12,13,14]. Considering this age-related difference, BC supplementation may affect the gut microbiome of young and aged mice differently. Therefore, in this study we used 3-month and 18-month old female mice, corresponding to the human ages of 20 and 56 years old, respectively. These ages are considered as mature adult and old, respectively [15]; and for the purposes of this study, the mature adult mice were referred to as “young” to contrast the 18-month old mice (“old”). After 4 months’ dietary intervention, they became 7 and 22 months old, which corresponds to the human ages of approximately 34 and 65 years old, respectively. We observed the microbiome composition changes in response to long-term BC supplementation and aimed to shed light on how BC supplementation exerts its health benefits through regulating the host gut microbiome.

## 2. Materials and Methods

### 2.1. Animals and Diets

This study was conducted concurrently with a recently published study on the effects of BC on bone mass among young and old female mice, in which the experimental design, chemicals and animal preparations were described [16]. Young (3 months old) and old (18 months old) female C57BL/6J mice (purchased from the Jackson Laboratory, Bar Harbor, ME, USA) were randomized to consume either a standard chow diet (AIN-93M diet from Dyets, Bethlehem, PA, USA) or a standard chow diet mixed with 1% (*w/w*) BC extract, which was provided by Just the Berries Ltd. (Palmerston North, New Zealand). As shown in Table 1, the BC extract contained not less than 30% of anthocyanins, and delphinidin-3-rutinoside and cyanidin-3-rutinoside were the major anthocyanin derivatives as analyzed in our previous research [5]. There were four groups, each with three mice: (**1**) Young Control, (**2**) Young BC, (**3**) Old Control, and (**4**) Old BC. After 4 months of feeding, individual fecal contents were collected from the colon directly and immediately stored in a −80 °C freezer for further processing. Body weight and food consumption were recorded weekly. All the procedures were approved by the Institutional Animal Care and Use Committee from the University of Connecticut and the University of Connecticut Health Center (protocol #100829-0117, approved 11/16/2015).

### 2.2. DNA Extraction PCR Amplification and Sequencing of Taxonomic Marker

Fecal samples were submitted to the University of Connecticut-Storrs Microbial Analysis, Resources and Services facility for microbiota characterization utilizing 16S V4 analysis. DNA was extracted from 0.25 g of fecal sample using the MoBio PowerMag Soil 96 well kit (Qiagen, Hilden, Germany) according to the manufacturer’s protocol for the Eppendorf epMotion liquid handling robot. DNA extracts were quantified using the Quant-iT PicoGreen kit (Invitrogen, ThermoFisher Scientific, Waltham, MA, USA). Partial bacterial 16S rRNA genes (V4, 0.8 picomole each 515F and 806R with Illumina adapters and 8 basepair dual indices (Kozich 2013)) were amplified in triplicate 15 uL reactions using GoTaq (Promega, Madison, WI, USA) with the addition of 10 µg BSA (New England BioLabs, Ipswich, MA, USA). To overcome initial primer binding inhibition because the majority of the primers do not match the template priming site, we added 0.1 femtomole 515F and 806R that did not have barcodes and adapters. The PCR reaction was incubated at 95 °C for 2 min, the 30 cycles of 30 s at 95.0 °C, 60 s at 50.0 °C and 60 s at 72.0 °C, followed by final extension as 72.0 °C for 10 min. PCR products were pooled for quantification and visualization using the QIAxcel DNA Fast Analysis (Qiagen, Hilden, Germany). PCR products were normalized based on the concentration of DNA from 350–420 bp then pooled using the QIAgility liquid handling robot. The pooled PCR products were cleaned using the Mag-Bind RxnPure Plus (Omega Bio-tek, Norcross, GA, USA) according to the manufacturer’s protocol. The cleaned pool was sequenced on the MiSeq using v2 2 × 250 base pair kit (Illumina, Inc., San Diego, CA, USA).

### 2.3. Sequence Data Processing

Sequences were demultiplexed using onboard bcl2fastq. Demultiplexed sequences were processed in Mothur v. 1.39.4 following the MiSeq SOP [17] Exact commands can be found here: https://github.com/krmaas/bioinformatics/blob/master/mothur.batch. Merged sequences that had any ambiguities or did not meet length expectations were removed. Sequences were aligned to the Silva nr_v119 alignment [18]. Taxonomic identification of operational taxonomic units (OTU)s was done to classify groups of closely related individuals using the Ribosomal Database Project Bayesian classifier [19] against the Silva nr_v119 taxonomy database.

### 2.4. Statistical Analysis

The OTU reads and taxonomy table were imported into R version 3.6.1 and all statistical analyses were performed using the phyloseq and vegan packages unless noted otherwise. The alpha diversity was measured by several diversity indices (observed species, Shannon diversity index, and inverse Simpson diversity index). A comparison was performed using two-way ANOVA. The beta diversity was assessed by weighted and unweighted UniFrac analysis. The difference was tested using PERMANOVA. Phylum abundance differences and OTU abundance differences among groups were evaluated by two-way ANOVA with post hoc comparisons (Bonferroni). The significance level for phylum difference was set at 0.05, while the OTU difference was set at 0.01.

## 3. Results

### 3.1. Age and BC Modified the Gut Microbial Community Composition

**Figure 1:**
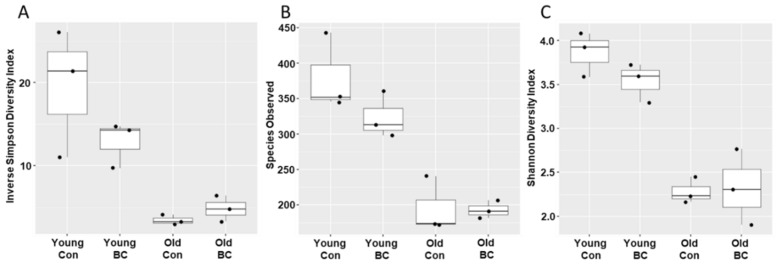
Alpha diversity of microbial samples based on age and diet. (**A**) Species observed, (**B**) Shannon diversity index, and (**C**) inverted Simpson diversity index. Data are represented as boxplots.

Discovered sequences were classified into 11 phyla, 26 classes, 46 orders, 74 families, and 116 genera. There were 1220 total OTUs discovered, and 719, 540, 344 and 330 OTUs in the young control, young BC, old control, and old BC groups, respectively (Figure 1). Old female mice had a less diverse microbial community composition compared to the young mice (Two-way ANOVA *p* < 0.0001 for species observed and Shannon diversity index; *p* = 0.001 for inverse Simpson diversity), whereas 4-month BC administration did not have a significant impact on their species richness (*p* = 0.21 for species observed, 0.41 for Shannon diversity index, and 0.31 for inverse Simpson diversity).

**Figure 2:**
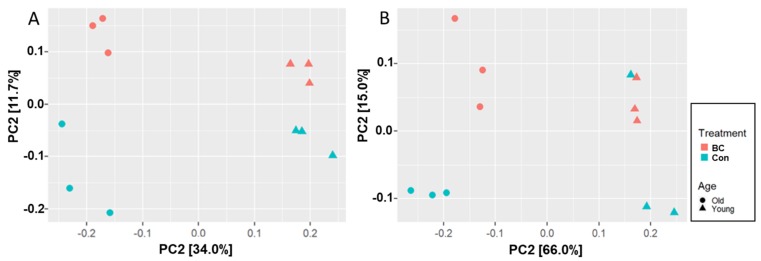
Principal coordinate analysis of microbial samples based on age and diet. (**A**) Unweighted and (**B**) weighted UniFrac distance. Young control (green triangle), young BC (orange triangle), old control (green circle), and old BC (orange circle).

In the beta diversity analysis (Figure 2), samples were well separated by age using both weighted UniFrac (PERMANOVA *p* value < 0.001) and unweighted UniFrac (PERMANOVA *p*  <  0.001). Samples with different diets were roughly separated using weighted UniFrac (PERMANOVA *p* = 0.06) and unweighted UniFrac (PERMANOVA *p* = 0.08). In both weighted and unweighted UniFrac, Principal coordinate 1 (PC1) (percent variation explained: 34% in unweight and 66% in weighted) separates samples with different ages, while PC2 (11.7% in unweighted and 15% in weighted) roughly separates the mice fed different diets.

### 3.2. Age and BC Modified the Phylum Composition in Mice

**Figure 3:**
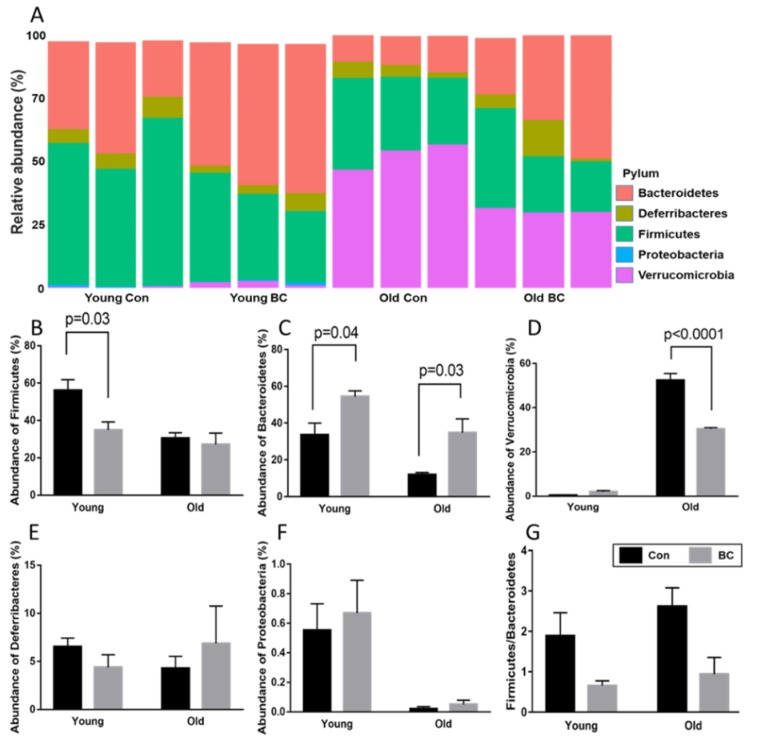
Phylum analysis of microbial samples with based on age and diet. (**A**) Composition of the six most abundant phyla in each sample. Relative abundance of individual phyla, including (**B**) Firmicutes, (**C**) Bacteroidetes, (**D**) Verrucomicrobia, (**E**) Deferribacteres, (**F**) Proteobacteria, (**G**) Ratio of fecal Firmicutes to Bacteroidetes in each group. Data are presented as mean ± SEM.

The phylum composition analysis is shown in Figure 3A. For clearer illustration, only the five most abundant phyla were included in this figure, which were Firmicutes, Bacteroidetes, Verrucomicrobia, Deferribacteres, and Proteobacteria. The abundance analysis of each phylum is shown in Figure 3B–G. According to the ANOVA results, several phyla showed an age-related decrease (*p* < 0.05), including Firmicutes, Bacteroidetes, Proteobacteria, and Tenericutes. In contrast, we saw an age-related increase of Verrucomicrobia (*p* < 0.0001). Exclusively of the genera *Akkermansia*, Verrucomicrobia phylum became the most dominant phylum in the old mice. BC also decreased the abundance of Verrucomicrobia in the old mice. Firmicutes, the most dominant phylum in the young mice, was suppressed by BC administration in the young mice but not in the old counterparts. The abundance of Bacteroidetes was increased by BC administration in both the young and old mice. After comparing the Firmicutes to Bacteroidetes (F/B) ratio, a significantly lower F/B ratio was seen in BC-fed mice than the control mice (*p* = 0.0093).

### 3.3. Age and BC Modified the Abundance of OTUs

There were 46 OTUs whose relative abundance differed between the young and old mice (*p* < 0.01; Table 2). Among the 46 OTUs, 40 decreased in old mice, while six increased in old mice. Twenty-three out of the 40 OTUs came from the *Lachnospiraceae* and *Rumicococcaceae* families. From the same families, three OTUs showed an age-related increase. Twelve OTUs from the Bacteroidales order had lower relative abundance in old mice than the young ones, while one OTU showed higher abundance in old than in young mice.

The relative abundance of eight OTUs were modified by BC supplementation (Table 3). Interestingly, all of them came from the *Lachnospiraceae* and *Ruminococcaceae* families. However, BC promoted the growth of four of them and suppressed the growth of the other four. After comparing the OTUs modified by age and the OTUs modified by BC, we found that only one OTU, from the family *Lachnospiraceae* changed by both BC and age. Aging modified the abundance of OTUs six times more than BC did, which was consistent with the patterns observed from the beta diversity analysis. These results indicate that age plays a paramount role in regulating gut microbiome.

There were 21 OTUs whose relative abundance had a significant interaction effect between BC and age (Table 4). To better illustrate the interaction between BC and age on gut microbiome, a heat map with the OTUs with significant interaction was created (Supplementary Figure S1). A majority of the 18 OTUs came from the families of *Lachnospiracear* and *Ruminococcaceae*. Of the 21 OTUs, 16 showed significant sensitivity to both BC and age. However, due to the significant interaction, they are not included in the previous tables.

## 4. Discussion

Anthocyanin-rich BC possesses antioxidant and anti-inflammatory properties that protect against certain chronic diseases such as cardiovascular disease, diabetes and neurodegenerative disease [1]. These health benefits are likely attributed to the anthocyanins’ interaction with the gut microbiome, at least in part. In vitro and in vivo studies showed that BC or bioactive compounds from BC promoted or inhibited the growth of certain bacterial species [3,20]. For instance, consumption of BC extract powder enhanced the growth of Lactobacilli and Bifidobacteria but suppressed the abundance of *Clostridium* spp. and *Bacteroides* spp. in healthy humans [21]. These studies, which focused on specific bacteria, provide evidence supporting BC’s regulation of the gut microbiome. Through investigating how the host gut microbiome changes in response to BC, we hope to have a better understanding of the health benefits of BC.

To our knowledge, this was the first study to compare the alpha diversity of the gut microbiome from young and old female mice. The alpha diversity analysis of the microbiome profile showed that old mice had a less diverse gut microbiome community. While we observed a remarkable decrease in alpha diversity in old female mice, others observed age-related increases in alpha diversity in males [22,23,24]. Previous studies have observed sex-specific differences in gut microbiome composition and response to diet intervention in hamsters and mice [25,26]. Taken together, it is readily apparent that sex should be considered when comparing results related to alpha diversity across sexes. The beta diversity analysis showed that aging plays a paramount role in regulating the structure of the gut microbiome, which has been reported previously [14,27]. The results also showed that the composition of the gut microbiome tended to differ in response to BC supplementation. However, this effect was not statistically significant in our study, which may be attributed to the small sample size.

In the young mice, BC supplementation significantly increased the abundance of the Bacteroidetes phylum while decreasing the Firmicutes phylum. Bacteroidetes and Firmicutes are two major phyla of gut microbiome [28]. The increased F/B ratio has been found to positively correlate with multiple chronic health disorders of the host, such as obesity, diabetes, and osteoporosis, although the mechanisms are unclear [29,30,31,32]. Well known health promoting phytochemicals such as green tea and black tea polyphenols may regulate host health through promoting the growth of Bacteroides while suppressing the growth of Firmicutes [33]. Another study also showed that pomegranate peel extract treatment for 14 days significantly reduced F/B ratio in mice [34]. In our study, we also observed a significantly lower F/B ratio in BC-fed mice than the control mice. Based on these abundance changes of Firmicutes and Bacteriodetes in the fecal sample, this study provides evidence that long-term BC supplementation alters the composition of the gut microbiome by reducing the F/B ratio, resulting in a gut environment that is more protective against certain chronic diseases.

Another interesting finding is that Verrucomicrobia phylum, exclusively of the genera *Akkermansia*, became the most dominant phylum in the old mice. BC also decreased the abundance of Verrucomicrobia in the old mice. The abundance change of phylum Verrucomicrobia with age was also seen in another female mouse study [14]. Furthermore, the enriched *Akkermansia* in the long-living groups compared to young counterparts was observed in humans [35]. The physiological role of Verrucomicrobia is not well studied. However, Verrucomicrobia was found to be more abundant in colonic content and feces from immunodeficient female Rag1^-/-^ mice compared to wild-type mice [36], which suggests that this phylum may be related to the compromised host immune status. Furthermore, members of this genus exacerbate gut inflammation in Salmonella Typhimurium-infected mice [37]. All these suggest that the increased abundance of this bacterium may indicate that the host is more vulnerable to inflammation. BC’s suppressive effect on Verrucomicrobia may relate with BC’s anti-inflammatory effect. In another study conducted with female mice, a high-fat diet also caused a sharp increase of Verrucomicrobia, which became the most dominant phylum [38]. Including our findings, it is noticeable that most Verrucomicrobia changes were observed in female hosts. The sex difference of Verrucomicrobia may be more critical than we expected.

When we analyzed the changes of microbiome at the OTU level, we found almost six times more OTUs modified by age than by BC. This is consistent with the beta diversity analysis, which showed a more profound effect from age than from BC on microbiome structure. The majority of the OTUs modified by age and all the OTUs mofied by BC were from two families, *Lachnospiraceae* and *Ruminococcaceae*. These two families are two of the most abundant families from the Clostridiales order in the mammalian gut environment and they serve as active plant degraders [39]. A study found that *Lachnospiraceae* and *Ruminococcaceae* abundance correlates with innate and acquired immunity [40]. These bacteria decompose the fiber, such as cellulose and hemicellulose components of consumed plant, and convert them into short chain fatty acids for absorption [39,40]. BC alters the composition of these fiber-degrading bacteria, which suggests that BC’s fiber content may play an important role in shaping the gut microbiome. However, different genera and OTUs showed different responses to age and BC. This suggests that the changes are more complicated than pure fiber-degrading; it may not be appropriate to combine and analyze the environmental effect at family level. The proliferative effects of the anthocyanins on *Bifidobacteria* (*B*. *bifidum*, *B*. *adolescents* and *B*. *infantis*) and *Lactobacillus* (*L*. *acidophilus*) under in vitro fermentation condition were reported recently [41]. However, we did not see any OTUs from these two genera change their abundance by BC supplementation. This may be due to the more complicated ecosystem in the host gut than the simple in vitro condition. Additionally, Bifidobacterial from the Actinobacteria phylum composed a very small fraction of the microbiome in our samples. The Lactobacillales order was also less predominant compared to the Cloristridiales order.

When we compared OTUs regulated by age and regulated by BC, only one OTU showed sensitivity to both factors. However, 21 OTUs showed a significant interaction between age and BC, among which 15 OTUs were also modified by both age and BC. This suggests that the gut microbiome responded differently to the BC supplementation in young and old mice, which corroborates with Zhu et al.’s findings that there are age-specific differences in gut microbiome in response to diet [42]. The food intake record showed a food intake difference between young and old mice in our previous study [16]. This result indicates that the supplemented BC intake was also different between young and old mice. Thus, the different intake of BC between young and old mice may also contribute to the 21 OTUs which showed significant interactions between age and BC.

Overall, the results suggest that long-term BC supplementation shapes the gut microbiome in female mice in an age-dependent manner. However, the limited sample size in this study makes the results exploratory in nature and future studies with larger sample sizes are needed to corroborate our discoveries. It is generally understood that the aging process changes the gut microbiome profiles; however, it is not well understood how dietary interventions affect the intestinal ecosystem. Future studies investigating the prebiotic function of foods such as BC should include populations from different ages and sexes to have a more comprehensive understanding of their benefits. Human hosts are also needed to provide more insight and relevance to humans on the microbiome-regulating effects of BC.

## Figures and Tables

**Table 1:** Nutrition facts and total anthocyanin contents of blackcurrant (*Ribes nigrum* L) extracts ^a^.

**Nutrition**	**% (1720 Kj/100g)**
Carbohydrate	89.2
Protein	3.9
Fat	3.8
Trans Fat	0.5
Saturated Fat	0.8
Ash	1.7
**Total anthocyanin composition**	**%**
Delphinidin-3-rutinoside	35–50
Delphinidin-3-glucoside	5–20
Cyanidin-3-rutinoside	30–45
Cyanidin-3-glucoside	3–10

^a^ The information was measured and provided by Just the Berries Ltd.

**Table 2:** Operational taxonomic units (OUT) abundance significantly modified by age ^a, b^.

Regulation	Phylum	Class	Order	Family	Genus	OTU	*p*-Value
Decreased	Firmicutes	Clostridia	Clostridiales	Lachnospiraceae	Lachnospiraceae_unclassified	Otu0045	0.0013
						Otu0047	0.0008
						Otu0059	0.0045
						Otu0061	0.0046
						Otu0074	0.0100
						Otu0089	0.0012
						Otu0124	0.0012
						Otu0125	0.0031
						Otu0201	0.0039
						Otu0314	0.0072
						Otu0351	0.0028
						Otu0353	0.0028
					Blautia	Otu0029	0.0049
						Otu0204	0.0036
						Otu0288	0.0046
					Acetatifactor	Otu0121	0.0016
					Coprococcus	Otu0141	0.0002
					Roseburia	Otu0069	0.0080
				Ruminococcaceae	Ruminococcaceae_unclassified	Otu0099	0.0013
						Otu0159	0.0007
						Otu0227	0.0005
					Oscillibacter	Otu0039	0.0003
						Otu0211	0.0034
	Bacteroidetes	Bacteroidia	Bacteroidales	Bacteroidaceae	Bacteroides	Otu0010	0.0000
						Otu0012	0.0053
						Otu0014	0.0014
						Otu0017	0.0024
						Otu0018	0.0079
						Otu0073	0.0000
						Otu0220	0.0085
						Otu0241	0.0063
				Porphyromonadaceae	Butyricimonas	Otu0006	0.0005
						Otu0134	0.0008
						Otu0333	0.0040
				Rikenellaceae	Alistipes	Otu0130	0.0003
		Bacteroidetes_unclassified	Bacteroidetes_unclassified	Bacteroidetes_unclassified	Bacteroidetes_unclassified	Otu0070	0.0074
	Cyanobacteria	Melainabacteria	Gastranaerophilales	Gastranaerophilales_unclassified	Gastranaerophilales_unclassified	Otu0020	0.0001
				Clostridiales_unclassified	Clostridiales_unclassified	Otu0064	0.0036
	Proteobacteria	Deltaproteobacteria	Desulfovibrionales	Desulfovibrionaceae	Desulfovibrio	Otu0184	0.0028
	Tenericutes	Mollicutes	RF9	RF9_unclassified	RF9_unclassified	Otu0181	0.0043
Increased	Firmicutes	Clostridia	Clostridiales	Lachnospiraceae	Lachnospiraceae_unclassified	Otu0036	0.0092
						Otu0156	0.0024
				Ruminococcaceae	Anaerotruncus	Otu0114	0.0001
		Erysipelotrichia	Erysipelotrichales	Erysipelotrichaceae	Incertae_Sedis	Otu0057	0.0007
	Bacteroidetes	Bacteroidia	Bacteroidales	Rikenellaceae	Alistipes	Otu0013	0.0027
	Verrucomicrobia	Verrucomicrobiae	Verrucomicrobiales	Verrucomicrobiaceae	Akkermansia	Otu0127	0.0029

^a^ Two-way ANOVA tests were performed to determine the main effect of age on individual OTUs. OTUs included are significantly modified (α < 0.01) by age, while interaction was not significant (α < 0.01); ^b^ OTU is used to classify groups of closely related individuals.

**Table 3:** OTU abundance significantly modified by BC ^a^.

Regulation	Phylum	Class	Order	Family	Genus	OTU	*p*-Value
Decreased	Firmicutes	Clostridia	Clostridiales	Lachnospiraceae	Lachnospiraceae_unclassified	Otu0053	0.0027
					Acetatifactor	Otu0058	0.0072
					Blautia	Otu0269	0.0012
				Ruminococcaceae	Incertae_Sedis	Otu0340	0.0028
Increased	Firmicutes	Clostridia	Clostridiales	Lachnospiraceae	Lachnospiraceae_unclassified	Otu0265	0.0068
					Lachnospiraceae_unclassified	Otu0156	0.0016
					Roseburia	Otu0142	0.0073
					Marvinbryantia	Otu0254	0.002

^a^ Two-way ANOVA tests were performed to determine the main effect of age on individual OTUs. OTUs included are significantly modified (α < 0.01) by long-term BC administration, while interaction was not significant (α < 0.01). OTU stands for operational taxonomic unit which is used to classify groups of closely related individuals.

**Table 4:** OTU abundance showed a significant interaction between age and BC ^a^.

Phylum	Class	Order	Family	Genus	OTU	*p*-Value		
						Age	BC	Age * BC
Firmicutes	Clostridia	Clostridiales	Lachnospiraceae	Lachnospiraceae_unclassified	Otu0025	0.0004	0.0006	0.0004
					Otu0119	0.0032	0.0037	0.0037
					Otu0123	0.0031	0.0018	0.0031
					Otu0143	0.2415	0.0578	0.0007
					Otu0217	0.6811	0.4186	0.0092
					Otu0290	0.0085	0.0085	0.0085
					Otu0332	0.0011	0.0011	0.0011
					Otu0433	0	0	0
				Incertae_Sedis	Otu0081	0.0446	0.0446	0.0036
					Otu0219	1E-05	0.0002	0.0002
					Otu0320	0.0085	0.0085	0.0085
				Blautia	Otu0050	0.0046	0.0017	0.0019
					Otu0190	0.0001	2E-05	0.0001
				Roseburia	Otu0118	0.0065	0.0026	0.0082
			Ruminococcaceae	Anaerotruncus	Otu0126	0.0001	0.0011	0.003
				Intestinimonas	Otu0152	0.0006	0.009	0.009
				Oscillibacter	Otu0221	0.0667	0.0222	0.0028
				Pseudoflavonifractor	Otu0092	0.1241	0.1149	0.0066
			vadinBB60	vadinBB60_unclassified	Otu0323	0.0805	0.0805	0.004
Verrucomicrobia	Verrucomicrobiae	Verrucomicrobiales	Verrucomicrobiaceae	Akkermansia	Otu0001	0	0.0002	7E-05
Bacteroidetes	Bacteroidia	Bacteroidales	Bacteroidaceae	Bacteroides	Otu0376	0.004	0.004	0.004

^a^ Two-way ANOVA tests were performed to determine the interaction between age and BC on individual OTUs. OTUs included showed a significant (α < 0.01) interaction between age and long-term BC administration. OTU stands for operational taxonomic unit which is used to classify groups of closely related individuals; * Indicates the interaction between two main factors (age and blackcurrant).

## Supplementary Materials

The following are available online at https://www.mdpi.com/2072-6643/12/2/290/s1, Figure S1: A heat map illustration with the OTUs about the interaction between BC and age on gut microbiome.
