# Supplementary figures and images for: Long-Term Blackcurrant Supplementation Modified Gut Microbiome Profiles in Mice in an Age-Dependent Manner: An Exploratory Study
Source: Nutrients. 2020 Jan 21;12(2):290. doi: 10.3390/nu12020290 (PMC7070352; doi:10.3390/nu12020290)

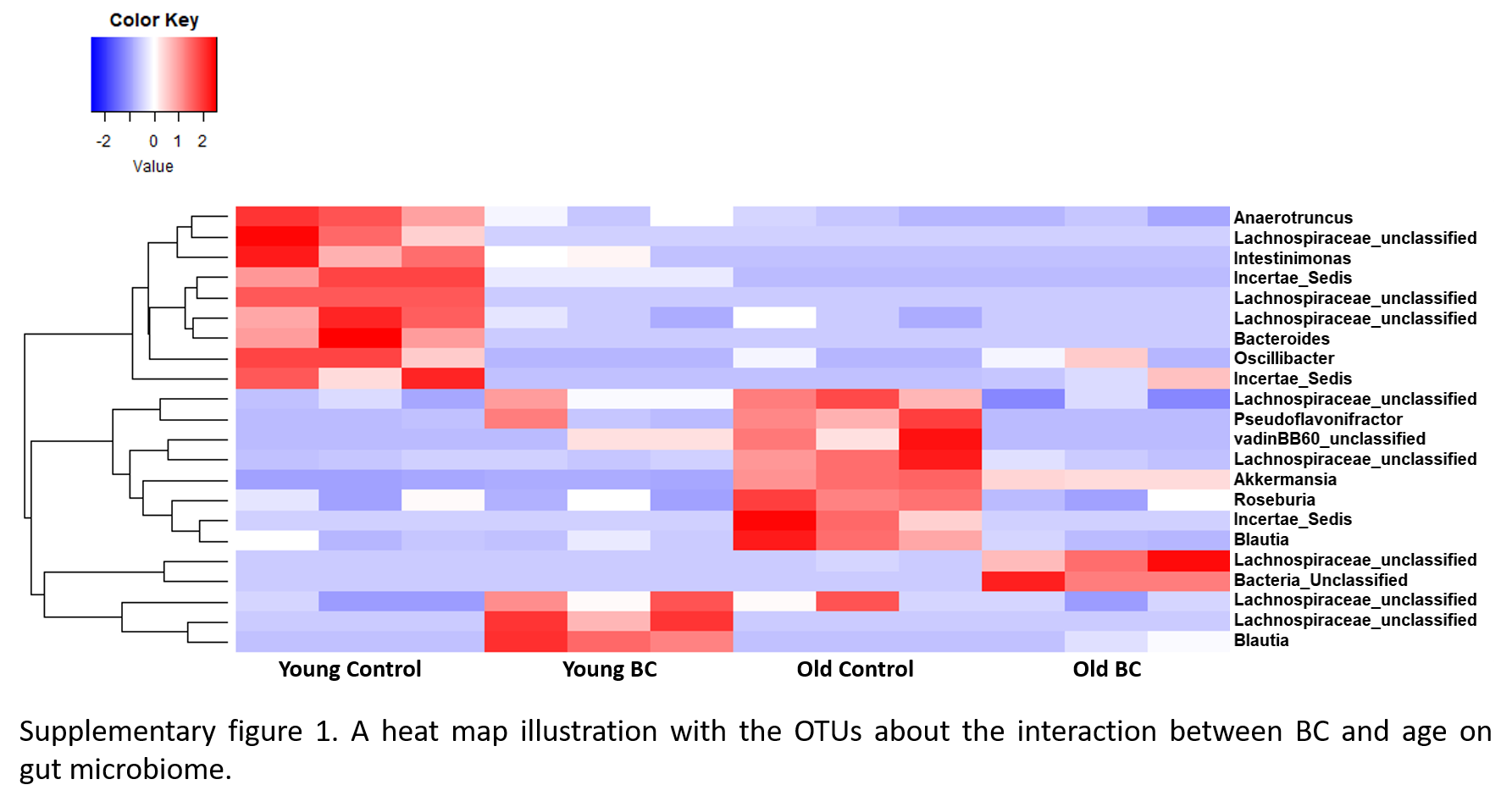

Supplement: Supplementary file 1 [file nutrients-12-00290-s001.zip › nutrients-680361-supplementary.tif]
